# Urban–Rural Differences in Long-Term Care Service Status and Needs Among Home-Based Elderly People in China

**DOI:** 10.3390/ijerph17051701

**Published:** 2020-03-05

**Authors:** Liangwen Zhang, Yanbing Zeng, Lixia Wang, Ya Fang

**Affiliations:** 1 State Key Laboratory of Molecular Vaccinology and Molecular Diagnostics, School of Public Health, Xiamen University, Xiamen 361102, China; liangwen_zhang@foxmail.com (L.Z.); ybingzeng@163.com (Y.Z.); alisiawang@163.com (L.W.); 2 Key Laboratory of Health Technology Assessment of Fujian Province University, School of Public Health, Xiamen University, Xiamen 361102, China; 3 School of Economics, Xiamen University, 422 Siming South Road, Xiamen 361006, China

**Keywords:** long-term care service, current status, needs, urban–rural differences, home-based elderly

## Abstract

Background: Long-term care (LTC) needs for the elderly have become increasingly crucial policy concerns in rapidly aging Asia, especially in China, the most populous nation. However, very few studies have examined the cohort differences in terms of their existing and expected utilization of LTC services, above all urban–rural differences. This study aims to evaluate the differences of LTC current status and needs between urban–rural areas and age groups, and to identify influencing factors causing the different LTC needs. Methods: The data come from the Chinese Longitudinal Health Longevity Survey in 2014. A total of 7192 home-based elderly aged ≥65 years by multistage sampling were enrolled. The Andersen Model was applied to categorize the influential factors into three components including predisposing, enabling and needs. Multivariate logistic regression analysis was used to analyze the influential factors of the three levels of LTC needs. Results: A total of 6909 valid sample sizes were included in this study. The overall LTC needs of the elderly showed a rapidly increasing trend among which older people had the highest needs for bathing (27.29%) and toileting (15.8%). It was also demonstrated the aged cohort between urban and rural exerted an impact on all aspects of LTC status and needs to varying degrees (*p <* 0.05). Compared with urban areas, the LTC needs for the elderly in rural areas was more vigorous, but the supply was seriously inadequate. The elderly who were older, living in rural areas, unmarried, non-farming, with low income, in poor health and having less autonomy had higher anticipated needs for LTC services (OR > 1, *p <* 0.01). Compared with the young-old in rural areas, the young-old in urban areas were prone to live alone (OR = 1.61, *p <* 0.01). The elderly who were older, living in rural areas, farming, with low income, lonely and depressed had higher anticipated needs for community-based services (1 *<* OR *<* 1.69, *p <* 0.05). Conclusions: The aged cohort in urban–rural distinction were facing an increasing need for immediate care due to the inadequate support being provided, especially among rural elderly. The oldest elderly in rural areas had higher LTC needs, and different levels of needs were affected by age, economic level, family support, health status and other related effects. This study provides evidence-based recommendation for further improving the construction and development of the LTC system in China.

## 1. Background

The aging population has become a serious social problem around the world, especially in China, the most populated nation. China has become an “aging society” as of 2000 and has experienced an unprecedented process in its aging population [1,2]. In 2017, there were 241 million people who were at least 60 years of age and 158 million who were at least 65 years of age in China, accounting for 17.3% and 11.4% of the total population, respectively [3]. It is being estimated that the proportion of individuals older than the ages of 60 and 65 in China will exceed 30% and 20%, respectively by 2050 [4]. The accelerated population aging has brought about health, social and economic issues, including decreased health function caused by disability and chronic diseases, declined labor supply and increased financial burden on long-term care (LTC) cost, social security, and individual savings [5]. China is undergoing profound socio-economic changes, and as the number of elderly people surges and the number of potential caregivers shrinks, Chinese families—traditionally the bedrock of elder care—are increasingly stressed [6]. As a result, formal LTC services for the elderly have emerged and expanded rapidly under the active promotion of the government’s public welfare policies in China.

As people get older, their homes and neighborhoods become even more important in their daily lives. Whether the society is traditional or modern, older people in China would prefer to age at home because of the traditional filial piety of Chinese traditional Confucian philosophy and the stigma of nursing homes that still exists [7,8,9]. Moreover, current policy and planning point out that home is the main site for people to age. In 2008, the government of Beijing Municipality proposed the “9073” elder care development strategy. The strategy calls for 90% of older people to age at home, 7% to age in place with government-purchased community service and 3% to age in institutions by 2020 [10]. Therefore, the main living space of the elderly, such as family, community and institutions for the aged, has increasingly become an important place to study China’s aging.

In 2007, the aging-friendly city was promoted by WHO, which was to create active aging, to let the elderly live in a comfortable environment and to achieve the goal of ‘aging in place’. The concept of “ageing in place” has been gaining attention in the recent years. In fact, the concept has been further defined to identify the concept of “remaining living in the community to have some more independence than in a residential care” [11]. Community services can be provided by integrating existing community resources with suitable local conditions. The social policy, with the emerging notions on ‘community care’ and ‘care in the community’, has been redefined in many aging societies to meet the needs of different levels of the elderly as well as to enable them to enjoy a healthy life in a familiar environment. This article highlights the concepts of active aging and aging in place, leading to the creation of policies and practices of gerontology to deal with the current diverse needs of the aging population in China. Furthermore, active aging is designed for the healthy older people, while aging in place is intended for the development of LTC policies for disabled seniors in China [12].

The Chinese government has recognized a series of problems and challenges brought about by the aging of the population. In recent years, a series of policies have been introduced to build and improve the long-term care systems for the elderly, especially in October 2015; during this time, the Communist Party of China announced the end of the decades-long one-child policy. Two children are now being allowed for each couple in China. One of the major purposes of the two-child policy is to deal with population aging, based on the statement from the Communist Party’s Central Committee. In addition, the government has established various national LTC schemes to meet the increasing LTC needs. Almost every pension policy formulated by the Chinese government has inevitably proposed to build an LTC system that is mainly based on family care, followed by community care and finally supplemented by institutional care [13,14]. Demographic changes in China have resulted in a great increase in the number of people with LTC needs. However, with this increase, families’ capability in providing LTC has been declining due to the former one-child policy which resulted in the massive outmigration of young people. In addition, the social security benefits and institutional care are not able to meet the needs of the frail and disabled people. In this case, the current care services provided by the family and community are obviously insufficient. Therefore, LTC is increasingly being recognized as a major social problem in China.

The LTC status and needs are important indicators in studying the distributional impacts of existing LTC policies. One of the key concerns in establishing a new LTC system is not being able to meet the needs of the rural and urban residents [15]. Since 1950, China has implemented a dual system of social welfare in urban and rural areas using the hukou system (a unique household registration system that originally institutionalized China’s rural–urban dual-society system) [16]. Due to the huge differences between urban and rural areas in terms of medical security, resource allocation, income levels, and living arrangements, the impact of LTC status and need among different elderly people in urban and rural areas may be also entirely different. A great amount of research in China’s healthcare system has been done to study the huge disparity existing in the healthcare use among the people in rural and urban areas [17,18]. While there is an increasing number of studies and reports in Western countries on LTC needs and inequalities in LTC use, little is still known about the LTC status and needs in the other parts of China [19,20]. Moreover, age is not only used to determine the population included in social security programs, but it is also used to identify need levels, average premiums and benefits [21]. Though less is known about the impact of age group and rural–urban disparities in LTC needs, information is indispensable when working in a more efficient and more equitable LTC system.

Studies on aging regarding current LTC needs in China now focus on exploring the contributing factors of different LTC modes or studying the impact of single-sex or urban–rural differences on LTC needs, most of which have a single research perspective. For instance, relevant literature has mainly explored the influencing factors of different LTC modes (also known as living arrangements) [22]. Some scholars describe the urban–rural differences both in the use and cost of LTC for the disabled elderly and explore potential influencing factors [23]. Some research focuses on the study of urban–rural inequality in the status of long-term care services [24]. In addition, related research defines LTC needs as a single two-category problem (inquiring whether the respondent has LTC requirements) and lacks a comprehensive evaluation of LTC needs [20,25]. Therefore, very few studies have examined the cohort differences in terms of their existing and expected utilization of LTC services (anticipated need for LTC services, anticipated living arrangement, anticipated community-based services), especially urban–rural differences.

In line with this context, the study on rural–urban disparities in relation to the LTC status and needs provides relevant information for the benefit of the health and LTC policy-makers. This process eventually triggers the implementation of new LTC programs. On one hand, LTC needs are significant in helping older individuals to better deal with their future needs and expenses. In the past decades, understanding the patterns of LTC status and needs of different cohorts, focused on urban–rural differences, is significantly important among policy makers behind the development of LTC services. So, this study aims to: (1) identify the current LTC situation and differences in needs of the urban and rural elderly at different age groups using nationally representative longitudinal data from China; (2) analyze the contributing factors causing the different LTC needs of the elderly, focusing on the urban–rural differences.

## 2. Methods

### 2.1. Sampling 

**Figure 1:**
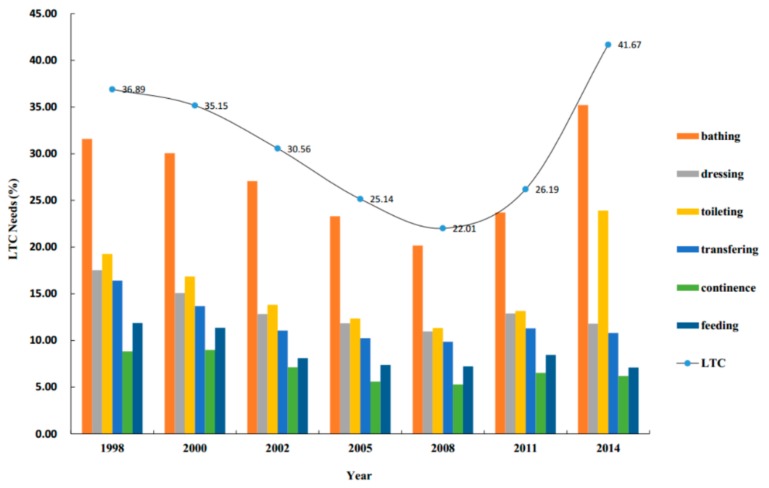
The status of LTC needs of the elderly from 1998 to 2014. Notes: LTC services needs including bathing, dressing, toileting, indoor transferring, continence and eating in this study. As long as any of the six daily living abilities of the elderly cannot be done and are indicated as needing need help, it means that the elderly have LTC needs.

We used data from the 2014 of the Chinese Longitudinal Healthy Longevity Survey (CLHLS) conducted by the Healthy Ageing and Development Research Center at Peking University, Beijing, China. The CLHLS was a high-quality, nationally representative survey on Chinese residents aged 65 years and above, with face-to-face interviews in respondents’ homes. It provided a large body of information on socioeconomic and demographic characteristics, health-related behaviors and lifestyles and LTC status. Besides, it included information on the care needs of the population with functional limitations as well as self-reported unmet LTC needs. We analyzed the status, needs and influencing factors of LTC for the elderly (Table 1, Table 2, Table 3, Table 4, Table 5 and Table 6) in 2014, and further used statistical charts (Figure 1) to see trends in the overall long-term care needs of the elderly from 1998 to 2014 (seven waves). Of all 7192 individuals in the 2014 survey, 283 cases were excluded due to living in LTC facilities (198 cases) or being less than 65 years old (85 cases); consequently, the sample consisted of 6909 home-based elderly people (3041 in urban areas, 3868 in rural areas).

### 2.2. Andersen Theoretical Model

In order to account for the variety of factors that may influence the LTC needs of the elderly, Andersen’s Behavioral Model of Health Services Use was adopted as the analytical framework. The Andersen model had been reported by several studies on elderly utilization of health services [26,27], health-related quality of life [28] and LTC services [29]. It showed the use of health services by predisposing (e.g., age, race, gender, marital status and education), enabling (e.g., family income, kinship network, social support network) and need components (e.g., health status, ADL). This theoretical analysis model better demonstrates the factors affecting LTC service needs of older people with different characteristics, but it is rare in the current literature. In this study, the Andersen model was used to analyze the associations of predisposing, enabling and needs variables with use of different LTC needs among the elderly.

#### 2.2.1. Dependent Variable

LTC needs were the central dependent variable, consisting of three dimensions [19], namely (1) anticipated need for LTC services, (2) anticipated living arrangement and (3) anticipated community-based services. The question measuring anticipated need for LTC services was phrased as: “Do you think you will need LTC services including bathing, dressing, toileting, indoor transferring, continence, eating?” Respondents were asked to answer the question using a three-point scale ranging from absolutely no need to partial need to completely needed. The anticipated future living arrangement was measured using the question: “What kind of living arrangement do you like best?” The answer options included: (1) living alone or with spouse no matter how far children live, (2) living alone or with spouse and children living nearby, (3) co-residence with children, (4) LTC institutions and (5) do not know. Respondents were asked to describe their expectations about their use of community-based services by seven items: “Do you expect your community to provide (1) personal care services, (2) home visits, (3) psychological consulting, (4) daily shopping, (5) social and recreational activities, (6) health education and (7) neighboring relations?” 

#### 2.2.2. Independent Variable

On the basis of the Andersen behavior model, this paper constructed the adjusted Andersen theoretical model of influencing factors on LTC needs according to the research purpose and variable information availability. The predisposing variables were age group (aged between 65 and 80 vs. aged 80+), gender, residence (urban vs. rural), marital status (married vs. unmarried), current living arrangement (whether or not living alone), educational level (years of schooling) and occupation (farmer vs. others). The enabling variables were expressed by the main source of financial support (retirement wages, salary, children and others), getting sufficient financial support to pay for daily expenses, family income (yes vs. no), economic status compared with other local people (poor, rich and fair), total household income last year (<15,000 yuan, 15,000–50,000 yuan, >50,000 yuan) and reported having medical insurance (medical insurance for urban workers, collective medical insurance for urban and rural residents, others).

Needs factors included ADLs (not limited, limited, strongly limited), numbers of chronic diseases (0, 1, 2 or more), the self-rated health (good, fair, bad), self-reported quality of life satisfaction (good, fair, bad), feeling fearful or anxious (seldom/never, always/often, sometimes), feeling lonely and isolated (seldom/never, always/often, sometimes) and feeling depressed (yes vs. no).

### 2.3. Analytical Methods

First, we divided the sample into four groups by age cohort and residence: (1) the young-old (aged 65 to 79) in urban areas, (2) the oldest old (age 80 and over) in urban areas, (3) the young-old (aged 65 to 79) in rural areas and (4) the oldest old (age 80 and over) in rural areas [30]. Then, descriptive statistics were presented for distribution characteristics of LTC status and needs among each subgroup mentioned above based on the Andersen Theoretical Model, while a chi-squared test was performed to examine significant differences for all dependent variables among the subgroups of respondents. A chi-squared or analysis of variance (ANOVA) was also performed to test significant differences for all independent variables. In addition, we performed binary and multinomial logistic regression to examine the multivariate relationship between each LTC need variable and predisposing, enabling and need characteristics. All statistical analyses were conducted using SAS 9.3. The odds ratio and 95% confidence interval indicated the effect of each predictor and whether it met statistical significance. A value of *p* < 0.05 was considered statistically significant.

## 3. Results

### 3.1. Predisposing, Enabling and Need Characteristics

A total effective sample size of 6909 individuals were included in this study, from 2014. The descriptive characteristics of the study sample are shown in Table 1. The predisposing, enabling and need characteristics were assessed by age cohort and residence. Of the sample (3856 females, 54.25%), the mean age was 85.62 ± 10.47 years; more than 40% of the elderly were married (41.01%); over half of them were not educated (56.51%); 7.11% lived alone; farmers accounted for 70.36%. In terms of the enabling variables, two-thirds (65.35%) of the elderly’s income was mainly from their children; 82% of respondents reported sufficient financial support to pay for daily expenses; 72.21% of them reported that economic status was fairly comparable with other local people; 96.37% had medical insurance (medical insurance for urban and rural residents accounted for 84.84%). All predisposing and enabling factors differed significantly in age group and residence, except living arrangement and economic status compared with other local people. Regarding needs factors, slightly more than one-tenth of respondents’ ADLs were strongly limited; 44.78% reported good subjective health status; 31.14% had at least two chronic illness; 67.08% reported good subjective quality of life; more than one-fifth of the elderly reported feeling lonely or isolated sometimes (22.40%). All needs factors differed significantly in age group and residence, except feeling fearful or anxious.

### 3.2. Status of LTC

Variables of LTC services status were assessed by age cohort and residence (Table 2). Nearly two-thirds of the elderly reported that they were mainly cared for by their children or grandchildren when they were sick or needed help, accounting for 67.44% and 68.49%, respectively. The majority (93.54%)of the elderly said that their primary caregivers were willing to take care of them. Additionally, 17.84% of the elderly said that the direct care cost last week was more than ¥500. Nearly 70% reported that the above-mentioned care costs were mainly paid by their children or spouses. Nearly two-fifths of the elderly reported that their primary caregivers had at least 35 h of care last week (18.53%). The proportions of the elderly to currently receive adequate medical services and treatment, were 96.05% and 71.97% respectively. In contrast, the community-based services available in the community were relatively scarce. Only one-third of the elderly reported that the community offered home visit and healthcare educational services, which accounted for 34.57% and 39.03%, respectively. Moreover, personal care, psychological consulting, and daily shopping services accounted for less than 10%. The age cohort in different residence had been found to exert an impact on all aspects of LTC status and needs to varying degrees, except for the care willingness of the primary caregiver (*p <* 0.05).

### 3.3. LTC Service Needs

LTC services needs and planning variables are assessed by age cohort and residence in Table 3. It is shown that almost half (41.67%) of the elderly reported their anticipated needs for LTC services in the future. As expected, the anticipated needs differed both in age cohort and in residence; the percentage was highest for the oldest old (aged 80+), especially in rural areas (54%), followed by the oldest old in urban areas and the young-old in rural areas, and it was lowest for the young-old in urban areas. Regarding their anticipated living arrangements, more than half of older people wanted to live with their children, while less than one percent of the older people wanted to live in an LTC facility. More than 60% of the oldest old in urban or rural areas were prone to live with their children. In terms of their anticipated community-based services, approximately 79.6% of respondents planned to use home visit, followed by healthcare education (73.34%) and psychological consulting (61.65%) or social and recreational activities (60.45%). Both for the young-old and oldest old, needs of community-based services in rural areas were higher than in urban areas.

From 1998 to 2014, the overall LTC needs of the elderly decreased first and then increased, among which older people had the highest needs for bathing and toileting, the average proportions of those being 27.29% and 15.80%, respectively. Especially in 2014, the total LTC needs of the elderly were as high as 41.67%. Of the sample, the needs for bathing and toileting among the elderly increased to 35.2% and 23.9%, respectively (Figure 1).

### 3.4. Anticipated Needs for LTC Services: Logistic Regression Analysis

Binary logistic regression was used to analyze factors associated with anticipated needs for LTC services. These results are presented in Table 4. A significance model of chi-squared statistics indicated that the independent variables examined reliably predict the expected LTC service needs (*R*^2^ = 0.23). Compared with the young-old in rural areas, the oldest old in urban or rural areas were more likely to express the need for LTC services (OR = 1.67, 4.16 *p <* 0.001) and the young-old in urban areas were less likely to seek the LTC services (OR = 0.51, *p <* 0.001). In addition, the elderly who were unmarried, non-farming, with low income, with poor health, and having less autonomy had higher anticipated needs for LTC services (OR > 1, *p <* 0.01).

### 3.5. Expected Future Living Arrangement: Multinomial Logistic Regression Analysis 

The results of the multinomial logistic regression analysis, examining correlations of anticipated future living arrangement, are shown in Table 5. The significance of the chi-squared model suggests that the independent variables reliably predict future living arrangement (*R*^2^ = 0.30). Compared with the young-old in rural areas, the young-old in urban areas were more likely to choose to live alone regardless of residential distance from children (OR = 1.61, *p <* 0.01). Moreover, older people who were male, married, not living alone, with low income, financially supported mainly by their children, with low ADLs, and living in a poor quality of life were more likely to choose to live with their children (0.23 < OR < 1, *p <* 0.05).

### 3.6. Expected Community-Based Service Needs: Logistic Regression Analysis

The third dependent variable, anticipated community-based service needs, was also regressed by three sets of independent variables, as can be seen in Table 6. Compared with the young-old in urban areas, there are more young-old people in rural areas who preferred to utilize social and recreational activities and healthcare educational services (OR = 1.27–1.32, *p <* 0.05), and more oldest old in rural areas planned to choose personal care, psychological consulting, social and recreational activities and healthcare educational services (OR = 1.21–1.33, *p <* 0.05). Living alone, occupation, financial support, loneliness and depression predicted the anticipated use of personal care and home visit (OR = 1.19–1.69, *p <* 0.05), while educational level, living alone, occupation and feeling lonely and isolated were significantly associated with the planned use of psychological consulting and daily shopping (OR = 1.20–1.64, *p <* 0.05). In short, those who were older, living in rural areas, living alone, higher educational level, farming, lower income, feeling lonely and depressed had higher anticipated needs for community-based services (1 *<* OR *<* 1.69, *p <* 0.05).

## 4. Discussion

Applying the Andersen Model to examine LTC expectations, the LTC status and needs of the young-old and oldest old cohorts of Chinese adults in urban and rural areas as well as their associated factors have been shown. Every residence and age cohort was examined individually by combining three aspects, which include anticipated LTC needs, living arrangement and community-based services. Birth-age cohort and residence have been found to have an impact on all aspects of LTC status and needs to varying degrees. The LTC needs for the elderly in rural areas was more vigorous, but the current situation of supply was seriously inadequate.

### 4.1. LTC Status

First and foremost, the study found that nearly two-thirds of the elderly in China were mainly cared for by their children or grandchildren when they were sick or needed help, and their primary caregivers were also willing to take care of them. Similar to the results of previous literature studies [31,32], the long-term caregivers for the elderly in China were their spouses and children, but with the decline of the spouse’s physical function and increase of chronic diseases, the role of their spouses in LTC was gradually weakened. Afterward, their children became the most important caregivers in practice, which was closely related to the filial piety in Chinese traditional Confucian philosophy. Several reports [33,34] have shown that the important role grandparents play in caring for grandchildren, unlike in Western countries; some described the children coming home from abroad to care for elderly parents and still others reported that amongst older Chinese people filial piety was an expectation and to be admitted to an elder care facility would be considered ‘a disgrace’. It can be seen that the concept of Chinese traditional filial care is deeply rooted. Chinese moral values, such as virtue and filial piety, are embedded in a Confucian moral and social context that cannot be altered without distortion in spite of Western and European notions [35]. The essay gives an emphasis on the Confucian resources being taken seriously in order to develop an authentic Chinese bioethics of LTC, as well as to come up with a defensive approach on LTC for contemporary society in general and the Chinese society in particular.

This study has shown that one-fifth of the elderly said that their direct care cost last week was more than ¥500. About 70% reported that the above-mentioned care costs were mainly paid by their children or spouses. The estimated annual direct care cost per elderly person was about 500 × 4 × 12 = 24,000 yuan, which was close to the national resident per capita disposable income (25,974 yuan) announced by the National Bureau of Statistics in 2017 [36]. In addition, almost two-fifths of the elderly reported that their primary caregivers spent at least 35 h in caring last week. According to the latest data from the National Bureau of Statistics in 2017, the national average annual wage is 74,318 yuan, about 20 yuan per hour. Therefore, the annual direct cost of human care for each elderly person is about 35 × 4 × 12 × 20 = 33,600 yuan [36]. It can be seen that whether it was direct care costs or human costs, the families of the elderly in China faced enormous financial risks and they could no longer afford the costs of LTC owing to the continuous deepening of disability or dementia of the elderly in some families; ultimately, the family care could no longer be sufficient to meet the elders’ needs. The long-term care insurance system that has been successfully implemented in many developed countries needs to be implemented as soon as possible in China to cope with the growing burden of care in family care.

This study indicated that the community-based services available in community were relatively scarce in general, especially in rural areas. Among them, personal care, psychological consulting and daily shopping services accounted for less than 10%. At present, the pension funds of our national community mainly rely on the central government and local financial investment, and the economic strength of the region and the government’s financial resources caused by the urban and rural two-dimensional structure make the community pension funds in urban and rural areas unevenly invested [37]. As the needs of old people in urban and rural communities gradually become more diversified, the structure of needs is diversified and the level of needs continues to grow. The content of community pension services is gradually showing defects such as single project, low level and narrow coverage [38]. Also, the overall development level of social work professionals in our community is not compatible with the needs of community pension services and faces problems such as urban–rural differences [39].

### 4.2. LTC Needs and Influencing Factors

The findings of this study suggested that the overall LTC needs of the elderly showed a rapidly rising trend from 2008 to 2014, among which are the older people with the highest needs for bathing and toileting. In accordance with the present results, previous ones have demonstrated that the overall prevalence of disability was highest in bathing (3.94%), followed by toileting (3.32%) [40]. A possible explanation behind might be that bathing is becoming a comprehensive and complex systemic movement. Similarly, the toileting also requires older people to have higher mobility, thus leading to a higher disability. In addition, the bathroom is the most vulnerable place for the elderly to fall, which will cause the elderly to lose their function again.

The most obvious finding from the analysis was that more than two-fifths of the elderly reported anticipated needs for LTC services in the future. It was found that the awareness level is relatively low in terms of LTC needs among the Chinese older adults; although 41.67 % of the respondents being reported has anticipated the LTC needs, the requested needs were still lower than what were found in a recent study made by Robison et al. (2014).The study indicated that two-thirds of the US respondents in their sample did expect the need for the LTC services [41]. This significant gap may be due to the lower awareness of LTC needs among Chinese older adults, while it could also be explained by their relatively good health status, compared to US peers. Furthermore, the most interesting finding was that there is a difference in the anticipated needs between age cohort and residence (*p <* 0.001). In terms of needs, the oldest elderly in rural areas had the highest percentage. The oldest elderly in urban areas and the young elderly in rural areas come in next, while the young elderly in urban areas had the lowest percentage. These results are likely to be related to the urban–rural differences in health status and healthcare system [17]. It has been shown in many previous studies that the older and rural older people are in poorer health status, which led to an increase in LTC needs [7,19]. In addition, although the healthcare system has greatly improved in rural areas, the health insurance program in rural China has not been as good (or beneficial) as the programs in urban areas. Older adults in rural areas also face obstacles in getting an ideal amount of care from family members (i.e., sons and daughters). This is a result of the increasing number of family members moving to urban areas for better education and work opportunities. As a consequence, the rural older adults get to face greater difficulties in getting adequate LTC services, compared to their urban counterparts.

By scrutinizing the descriptive results, the predisposition of variables, in line with the capabilities and needs among residence and age cohort, will be made clearer especially in understanding the results shown above. In this study, the elderly who were unmarried, with low income, with poor health and with less autonomy had higher anticipated needs for LTC services. One previous study showed a link between social support and LTC needs. Weak social support tends to generate higher LTC needs [42]. Unmarried seniors with low income have weaker family support and socio-economic support, which has led to higher long-term care needs, especially in rural areas. Additionally, in the conceptual model, there is an indicator showing the need of people with disability. In another study, it was found that people whose health are at worse or far more chronic conditions were less likely able to make some plans [43], However, with this population, there was an increase of expectations among disabled persons who are in need of the LTC services. However, this result has not previously been described. One interesting finding was that older people with less autonomy had higher anticipated needs for LTC services. The study showed that those living in private households were with worse mental health than the residents in institutional homes. It is suggested that the long-term care environment can constrain the elder’s autonomy, which leads to depression [44]. Depression may lead to a series of serious physical or mental illness, which could cause the further long-term care needs of the elderly.

As expected, regarding their anticipated living arrangements, more than half of older people wanted to live with their children, while less than one percent of older people wanted to live in an LTC facility. More than 60% of the oldest old in urban or rural areas tended to live with their children. Similar to the findings reported by Robison et al. (2014) [41], the results revealed that there is a greater preference for ageing in place among Chinese older adults, which shows similar results with another study indicating that almost three-quarters of the elderly in Hong Kong agreed or strongly agreed that ‘receiving care at home is better than that at residential facilities’ [20]. Although there is an increasing possibility for older adults with chronic disabilities to age in the community rather than in institutional settings, the supportive living arrangements must be provided in advance. Home modification is therefore needed for safety purposes at home or for creating a wheelchair pathway. Unlike the aging process or a life-long intellectual disability, neither of which can be reversed, the physical environment can be modified to ameliorate the negative impacts of both aging and disability. More modifications were made in the bathroom than in any other areas of the home [45]. Changes may include adding grab bars and replacing towel racks with these bars to prevent further accidents among the elderly, adding non-slip mats on the bathroom floor, installing anti-scald devices on faucets with adjustable height, hand-held showerheads, improving lighting in the shower and lowering medicine cabinets that were out of reach.

The results of this study indicate that the young-old in urban areas were more likely to choose to live alone regardless of residential distance from children compared with the young-old in rural areas. On the one hand, the health status of young urban residents may be better than that of rural peers. They often have a high level of education and income and the ability to receive new things [46]. Moreover, choosing to live alone in the case of perfect physical function can reduce the burden of care on children. On the other hand, this also shows that the living arrangements of rural elderly are particularly ingrained by the influence of traditional Chinese filial piety culture [47]. In addition, reliance on family support for care needs is likely not able to diminish, and older people who were married, not living alone, with low-income, getting financial support mainly provided by children, with low ADLs and living in a poor quality of life may in reality choose to live with their adult children more often. Many predisposing, capabilities, and needs factors previously identified in the literature as correlates of LTC planning indicate the findings within expectation, whereas others do not. These results are in accordance with recent studies indicating that the more sophisticated the family care support system that older people receive, the more likely they prefer to choose to live with their children. For example, the elderly in this study are married, not living alone, getting financial support for children, etc. However, this finding is contrary to previous studies which have suggested that the elderly with low ADLs and living in poor quality of life were more likely to choose living LTC facilities [7]. This study found that the worse the physical condition of the elderly, the more they choose to live with their children. The reason may be related to the sample of this study, which was taken from a national sample in 2014. At that time, the number of institutions in China was low and the quality of service was not perfect. The elderly still had a large misunderstanding of institutional care. Therefore, at present, the proportion of elderly people who choose institutions in this study is likely to be underestimated.

In terms of anticipated community-based services, a variety of these were supported by the respondents in this study, with a home visit, healthcare education and psychological consulting being the most needed, especially in rural areas. The young-old almost in all categories represented higher needs than the oldest elderly. A recent study from Hong Kong also confirmed that the young-old cohort in China was associated with higher educational levels and better financial literacy; more savings, income and assets; and higher social class compared with oldest elderly. All of these factors may raise their expectations for future LTC service needs while financially enabling them to handle various aspects of these services as well [20]. Additionally, fragmented health insurance schemes are inextricably linked to the existing gaps between urban and rural areas like in the difference of benefits and reimbursement levels, causing inequitable healthcare utilization and health outcomes. Evidence also shows that the current health insurance schemes have failed to reduce the inequality existing in the income of the elderly patients, who are also in great need of LTC services whether they are in urban or rural areas. In the previous studies, it was argued that the association between unmet need and social deprivation strengthens at higher levels. Therefore, the elderly in the rural areas face a higher risk of experiencing a shortage for caregivers because of the heavy outmigration of adults and a larger increase in the older aged population compared to the urban group [6], which to some extent further exacerbates the disparities of the LTC needs between rural and urban areas.

This study found that the young-old in urban areas were more willing to utilize social and recreational activities and healthcare educational services; the oldest old in rural areas planned to choose personal care and psychological consulting in addition to the above two, compared with the young-old in urban areas. On one hand, caregivers are facing increasing needs to carry out complex daily social, healthcare, education and personal care and also to take on nursing tasks and household chores for their families with chronic illnesses and disabilities. Traditional family informal care is far from meeting the multi-level care needs of the current elderly, and there is a strong need for formal community home care services [10]. On the other hand, the young-old in rural areas are mostly empty nesters, with a strong sense of loneliness, and may have higher needs for multi-level LTC services, which in turn has created a high needs for personal care and counseling [23]. Furthermore, we found that the elderly who were older, living in rural areas, living alone, of higher educational level, farming, with lower income, lonely and depressed had higher anticipated needs for community-based services. Consistent with the results from the USA (Black, Reynolds and Osman 2008), higher educational levels of elderly led to greater needs in using community-based services [43]. The elderly’s level of limitation in ADL and feeling lonely and depressed were the most distinguishing characteristics for identifying older adults who failed to take up their referred community-based services [48]. First, the objective reason may be that older people with poor health are more willing to accept LTC services. Secondly, the elderly with a higher educational level are more comprehensive in their knowledge of health care and rehabilitation, as well as willing to try and accept relatively innovative care services, subjectively.

However, there are several limitations existing in this study. First, the data in our study came from a cross-sectional survey and this confined the interpretation of our results, making it hard to draw causal conclusions. It is recognized that longitudinal data are needed to further explore the causal and temporal relations between the factors examined and LTC needs. Second, due to the limitation of data, this study lacks research on the needs for long-term care insurance for the elderly. In the future, we will supplement the long-term care insurance arrangement for the elderly as an important research content.

## 5. Conclusions

Since little is known about the older adults in planning for their future LTC needs, policymakers and planners designed a service support structure and residential areas without sufficient knowledge of their clients’ or constituents’ potential behavior. There are assumptions that the older adults will have the same kind of support needs and residential preferences in rural and urban areas, as the current cohort may possibly lead to inadequate system-wide planning. This study is the piece which fills that missing part of the puzzle, by examining the potential influence of age cohort and residence on the LTC needs on a large scale. The aged cohort in urban–rural distinction were facing an increasing need of immediate care due to the inadequate supports being provided, especially among rural elderly. The oldest elderly in rural areas had higher LTC needs, and different levels of needs were affected by age, economic level, family support, health status and other related effects. Thus, some implications for policy-making can be drawn based on this study to inform the development of LTC services in China.

First and foremost, the state should vigorously develop community care services, especially in rural areas, and improve the LTC service system which integrates family care, institutional care and community care as soon as possible. Based on the merits and demerits of family care and institutional care, it is emphasized that the elderly receive aging in place and that the community-based home care model is vigorously developed. Second, community day-care services should be rich in content, diversified in service forms and focus on urban and rural differences. It is recommended to organize professional training in nursing for family members who will provide long-term care for the disabled elderly by social workers as the core, by employing professional medical and nursing personnel. Community pension centers should be staffed with professionals who are responsible for day care, rehabilitation, psychological comfort and cultural and recreational activities for the elderly, who are either returned by their families or sent home by the center at night. Also, policymakers need to develop an LTC insurance system as soon as possible, to provide service subsidies for families with severe financial difficulties and disabilities, focusing on rural areas.

## Figures and Tables

**Table 1:** Predisposing, enabling and need characteristics by age cohort and residence.

Variables	*n* (%)	Urban		Rural	
		Aged <80	Aged 80+	Aged <80	Aged 80+
Predisposing Variables:					
Age ***					
Mean	85.62	74.30	90.98	73.28	91.60
Range	65–117	60–79	80–115	60–79	80–117
Gender * (female)	3876 (54.25)	542	1115	536	1588
		Percentages			
Married (yes) ***	2785 (41.01)	66.73	27.95	70.45	25.28
Years of schooling (vs. No education) ***					
1–7	2192 (31.63)	43.49	29.20	42.73	22.82
>7	822 (11.86)	25.20	10.14	19.25	3.76
Living alone (yes)	425 (7.11)	7.01	6.05	6.43	8.40
Occupation (farmer) **	4469 (70.36)	54.38	57.41	81.52	82.89
Enabling variables:					
Main source of financial support (vs. Others)***					
Retirement wages/salary	1848 (27.10)	50.32	28.56	39.75	9.72
Children	3653 (65.35)	30.73	50.69	44.44	70.16
Financial support sufficient to pay for daily expenses (yes)*	5590 (82.02)	82.48	84.40	79.60	81.20
Economic status compared with other local people (vs. Poor)					
Rich	1104 (16.26)	17.74	21.66	13.70	12.72
Fair	4902 (72.21)	71.61	67.68	73.79	75.17
Total income of your household last year (vs. ¥0–15,000) (yuan) ***					
¥15,000–50,000	2200 (34.52)	40.17	36.24	34.30	30.84
>¥50,000	1545 (24.24)	24.78	32.01	18.56	16.92
Has medical insurance support (vs. Others)***		71.61	67.68	73.79	75.17
Medical insurance for urban workers	761 (11.95)	26.03	19.40	6.17	3.37
Collective medical insurance for urban and rural residents	5376 (84.42)	69.84	75.65	91.12	93.73
Needs variables: ADLs (vs. Not limited) ***					
Yes, strongly limited	860 (12.54)	7.55	17.89	4.81	14.45
Yes, limited	1478 (21.55)	15.27	25.51	15.21	24.40
Self-rated health (vs. Bad) *					
Good	2871 (44.78)	45.40	45.03	48.22	42.41
Fair	2556 (39.87)	39.52	38.73	36.74	42.63
Numbers of chronic illness (vs. 0)***					
1	2109 (30.15)	26.59	29.43	32.47	31.05
2 or more	2178 (31.14)	45.28	34.27	27.87	24.49
Self-reported quality of life (vs. Bad) ***					
Good	4304 (67.08)	66.48	71.66	62.36	66.45
Fair	1871 (29.16)	30.76	25.17	33.84	28.90
Feel fearful or anxious (vs. Seldom/never)					
Always/often	228 (3.68)	3.69	3.31	3.99	3.77
Sometimes	1390 (22.40)	17.99	18.85	24.82	25.98
Feel lonely and isolated (vs. Seldom/never) ***					
Always/often	432 (6.94)	4.88	6.82	6.62	8.24
Sometimes	1533 (24.63)	17.60	23.93	22.27	30.02
Make own decision (vs. Seldom/never) ***					
Always/often	3730 (60.89)	72.61	62.21	68.48	49.56
Sometimes	1392 (22.72)	18.29	21.82	20.61	26.88
Feel depressed ^#^ (yes) **	677 (11.73)	13.57	13.63	13.01	8.59

Note: *n* = 6909. ^#^ Depressed: felt depressed for two weeks or more in the last 12 months; Differences between groups were tested by an analysis of variance or chi-squared test, as appropriate. * *p <* 0.05, ** *p <* 0.01, *** *p <* 0.001.

**Table 2:** Status of long-term care (LTC) service among the elderly by age cohort and residence.

Variables	*n* (%)	Urban (%)		Rural (%)	
		Aged <80	Aged 80+	Aged <80	Aged 80+
Primary caregiver when assistance needed (vs. others) ***					
spouse	384 (16.45)	30.13	11.59	39.20	11.61
children/grandchildren	1574 (67.44)	32.22	74.24	36.80	77.93
Who takes care of you when you are sick (vs. others) ***					
spouse	1179 (27.50)	48.48	15.42	48.11	16.01
children/grandchildren	2936 (68.49)	46.85	80.37	47.16	81.22
The willingness of the primary caregiver (vs. unwilling)					
willing to do	1810 (93.54)	92.47	94.04	93.26	93.33
without patience	28 (1.55)	1.37	1.17	1.12	1.79
need respite care	75 (4.14)	4.79	2.98	3.37	4.65
Total direct cost paid for caregiving last week (vs. 0) (Yuan) **					
¥1–500	721 (41.63)	40.80	38.51	38.31	45.31
>¥500	309 (17.84)	18.40	24.71	7.79	13.47
Who mainly pays the above cost (vs. others) ***					
self/spouse	361 (21.99)	52.14	23.56	39.86	12.42
children and their spouses	1141 (69.49)	41.88	66.15	54.55	79.62
Hours did the primary caregiver help last week(vs.0) ***					
1–35	1661 (37.72)	27.58	40.28	30.89	42.53
>35	816 (18.53)	6.66	28.07	5.06	21.38
Get adequate medical service at present(yes) ***	6549 (96.05)	98.17	96.04	96.67	94.85
Got adequate medical treatment at present (yes) ***	4713 (71.97)	93.79	88.17	93.24	83.35
Available community-based services					
personal care (yes)	269 (3.99)	5.50	4.99	2.91	3.12
home visit (yes) ***	2341 (34.57)	30.82	33.57	33.12	37.69
psychological consulting (yes) *	540 (8.01)	11.36	9.60	6.86	5.92
daily shopping (yes)	696 (9.35)	8.36	7.49	13.41	11.88
social and recreational activities (yes) *	1140 (16.95)	21.93	19.31	14.85	14.00
legal aid (yes) *	839 (12.49)	17.52	14.12	10.27	10.15
healthcare education (yes)	2634 (39.03)	41.68	38.84	39.13	37.97
neighborhood relation (yes) *	1676 (24.94)	31.35	28.96	21.86	20.56

Note: *n* = 6909. * *p* < 0.05, ** *p* < 0.01, *** *p* < 0.001.

**Table 3:** LTC service needs by age cohort and residence.

Variables	*n* (%)	Urban (%)		Rural (%)	
		Aged <80	Aged 80+	Aged <80	Aged 80+
Anticipated need for LTC services (yes) ***	2879 (41.67)	8.80	36.91	13.78	54.00
Anticipated living arrangements ***					
Living alone regardless residential distance from children	910 (13.01)	22.82	10.75	21.49	10.34
Living alone (or with spouse) and children living nearby	2039 (29.15)	38.22	26.84	39.54	27.96
Co-residence with children	3979 (56.89)	38.12	61.68	37.92	60.67
LTC facility	66 (0.94)	0.85	0.73	1.05	1.03
Anticipated community-based services					
Personal care	3919 (56.03)	54.83	57.49	60.11	59.44
Home visit ***	5567 (79.60)	76.45	79.63	83.48	86.47
Psychological consulting	4312 (61.65)	61.93	63.99	65.45	64.56
Daily shopping	3725 (53.26)	51.40	53.89	58.56	57.12
Social and recreational activities **	4228 (60.45)	64.72	62.09	65.59	63.69
Legal aid **	4024 (57.54)	60.34	58.84	64.12	59.19
Healthcare education **	5199 (74.34)	77.65	75.21	81.52	76.94
Neighborhood relation **	4236 (60.57)	62.58	61.24	68.56	62.52

Note: *n* = 6909. * *p <* 0.05, ** *p <* 0.01, *** *p <* 0.001.

**Table 4:** Odds ratios of the anticipated needs for LTC services (binary logistic regression).

Variables	Anticipated Need for LTC Services
Predisposing variables:	
Group (Ref. Rural aged < 80)	
Urban aged < 80	0.51 (0.35,0.74) ***
Urban aged 80+	1.67 (1.30,2.13) ***
Rural aged 80+	1.46 (1.31,2.66) ***
Gender (Ref. Male)	0.99 (0.77,1.26)
Married (Ref. No)	0.54 (0.42,0.70) ***
Years of schooling (Ref. 0)	
1–7	0.87 (0.67,1.14)
>7	0.83 (0.55,1.26)
Living alone (Ref. No)	0.32 (0.20,0.53) ***
Occupation (Ref. Non- farming)	0.58 (0.44,0.75) ***
Enabling variables:	
Main source of financial support (Ref. Others)	
Retirement wages/salary	0.69 (0.46,1.02)
Children	1.12 (0.86,1.46)
Financial support sufficient to pay for daily expenses (Ref. No)	0.80 (0.58,1.10)
Economic status compared with other local people (Ref. Poor)	
Rich	0.84 (0.53,1.33)
Fair	0.66 (0.45,0.98) *
Total income of your household last year (Ref. <¥15,000) (Yuan)	
¥15,000–50,000	0.95 (0.73,1.23)
>¥50,000	0.64 (0.47,0.86) **
Has medical insurance support (Ref. Others)	
Medical insurance for urban workers	0.69 (0.38,1.26)
Collective medical insurance for urban and rural residents	0.56 (0.35,0.91) *
Needs variables:	
Self-rated health (Ref. Bad)	
Good	0.92 (0.66,1.28)
Fair	0.78 (0.58,1.05)
Numbers of chronic illness (Ref. 0)	
1	1.42 (1.1,1.85) **
2 or more	1.28 (0.99,1.67)
Self-reported quality of life (Ref. Bad)	
Good	1.21 (0.66,2.23)
Fair	0.98 (0.54,1.78)
Feel fearful or anxious (Ref. Seldom/never)	
Always/often	1.08 (0.63,1.86)
Sometimes	0.93 (0.70,1.24)
Feel lonely and isolated (Ref. Seldom/never)	
Always/often	0.47 (0.29,0.77) **
Sometimes	0.90 (0.69,1.18)
Make own decision (Ref. Seldom/never)	
Always/often	0.57 (0.43,0.74) ***
Sometimes	0.63 (0.47,0.86) **
Feel depressed ^#^ (Ref. No)	0.85 (0.62,1.17)
	
Model summary:	
Nagelkerke R^2^	0.23
χ2 with df = 32 (*p*-value)	957.08 (<0.0001)
−2Log likelihood	2484.80

Note: *n* = 6909. Ref.: reference category. Taking “no anticipated needs for LTC services” as a reference. Values in parentheses are the odds ratios without controlling for other variables. * *p* < 0.05, ** *p* < 0.01, *** *p* < 0.001.

**Table 5:** Odds ratios of anticipated living arrangements (multinomial logistic regression).

Variables	Living Alone ^1^	Living Alone ^2^
Predisposing variables:		
Group (Ref. Rural aged <80)		
Urban aged <80	1.61 (1.14,2.28) **	1.28 (0.97,1.68)
Urban aged 80+	0.95 (0.68,1.32)	1.05 (0.82,1.33)
Rural aged 80+	1.19 (0.85,1.67)	1.18 (0.91,1.53)
Gender (Ref. Male)	0.75 (0.57,0.97) *	0.93 (0.76,1.14)
Married (Ref. No)	6.68 (4.90,9.10) ***	5.6 (4.52,6.95) ***
Years of schooling (Ref. 0)		
1–7	1.00 (0.76,1.32)	0.97 (0.79,1.20)
>7	0.99 (0.69,1.44)	0.89 (0.65,1.20)
Living alone (Ref. No)	8.44 (5.34,13.33) ***	7.38 (5.16,10.56) ***
Occupation (Ref. Non-peasant)	0.65 (0.48,0.88) **	0.83 (0.65,1.05)
Enabling variables:		
Main source of financial support (Ref. Others)		
Retirement wages/salary	0.91 (0.65,1.28)	0.99 (0.75,1.31)
Children	0.47 (0.34,0.64) ***	0.63 (0.50,0.80) ***
Financial support sufficient to pay for daily expenses (Ref. No)	0.74 (0.52,1.05)	0.66 (0.51,0.86) **
Economic status compared with other local people (Ref. Poor)		
Rich	1.05 (0.63,1.75)	1.55 (1.03,2.33) *
Fair	0.98 (0.64,1.49)	1.34 (0.95,1.89)
Total income of your household last year (Ref. <¥15,000)		
¥15,000–50,000	0.39 (0.30,0.52) ***	0.45 (0.37,0.56) ***
>¥50,000	0.23 (0.16,0.32) ***	0.24 (0.19,0.31) ***
Has medical insurance support (Ref. Others)		
Medical insurance for urban workers	0.97 (0.51,1.86)	1.47 (0.85,2.54)
Collective medical insurance for urban and rural residents	0.60 (0.34,1.05)	0.96 (0.60,1.55)
Needs variables:		
ADLs (Ref. Not limited)		
Yes, strongly limited	0.58 (0.35,0.95) *	0.95 (0.68,1.32)
Yes, limited	1.07 (0.79,1.45)	1.21 (0.97,1.51)
Self-rated health (Ref. Bad)		
Good	0.95 (0.64,1.42)	0.99 (0.73,1.34)
Fair	0.87 (0.6,1.25)	0.91 (0.68,1.20)
Numbers of chronic illness (Ref. 0)		
1	1.15 (0.87,1.51)	0.96 (0.78,1.19)
2 or more	1.02 (0.77,1.35)	1.05 (0.85,1.31)
Self-reported quality of life (Ref. Bad)		
Good	2.36 (1.07,5.19) *	1.81 (1.00,3.27)
Fair	2.53 (1.17,5.51) *	1.77 (0.99,3.18)
Feel fearful or anxious (Ref. Seldom/never)		
Always/often	1.51 (0.80,2.88)	1.26 (0.75,2.11)
Sometimes	0.88 (0.63,1.23)	1.14 (0.89,1.45)
Feel lonely and isolated (Ref. Seldom/never)		
Always/often	1.34 (0.78,2.28)	1.20 (0.78,1.83)
Sometimes	1.00 (0.72,1.39)	1.11 (0.87,1.42)
Make own decision (Ref. Seldom/never)		
Always/often	1.86 (1.31,2.65) ***	1.69 (1.30,2.19) ***
Sometimes	0.68 (0.44,1.05)	1.15 (0.86,1.54)
Feel depressed ^#^ (Ref. No)	0.67 (0.47,0.97) *	0.98 (0.73,1.31)
Model summary:		
Nagelkerke *R*^2^	0.30	
χ2 with df = 64 (*p*-value)	1297.17 (<0.001)	
−2Log likelihood	5745.14	

Note: *n* = 6909. Taking “Co-residence with children” as a reference. Values in parentheses are the odds ratios without controlling for other variables. Living alone ^1^: Living alone regardless residential distance from children; Living alone ^2^: living alone (or with spouse) and children living nearby. * *p <* 0.05, ** *p <* 0.01, *** *p <* 0.001.

**Table 6:** Odds ratios of anticipated community-based services (binary logistic regression).

Variables	Personal Care	Home Visit	Psychological Consulting	Daily Shopping	Social and Recreational Activities	Legal Aid	Healthcare Education
Predisposing variables: Group (Ref. Urban aged <80)							
Rural aged <80	1.12 (0.90,1.39)	0.62 (0.47,0.82) ***	1.34 (0.91,1.42)	0.97 (0.78,1.20)	1.27 (1.02,1.58) *	1.13 (0.91,1.41)	1.32 (1.02,1.70) *
Rural aged 80+	1.21 (1.01,1.45) *	0.83 (0.65,1.06)	1.33 (1.10,1.60) **	1.11 (0.93,1.33)	1.27 (1.05,1.52) *	1.12 (0.93,1.34)	1.25 (1.01,1.54) *
Urban aged 80+	1.12 (0.91,1.38)	0.75 (0.57,0.99)*	1.07 (0.86,1.32)	0.95 (0.77,1.16)	1.14 (0.92,1.40)	1.12 (0.90,1.38)	1.29 (1.01,1.66) *
Years of schooling (Ref. 0)							
1–7	1.16 (0.98,1.37)	0.95 (0.76,1.17)	1.11 (0.94,1.32)	1.20 (1.01,1.42) *	1.16 (0.97,1.37)	1.13 (0.95,1.34)	1.17 (0.95,1.43)
>7	1.26 (0.99,1.61)	0.85 (0.63,1.14)	1.19 (0.93,1.53)	1.52 (1.19,1.94) ***	1.31 (1.02,1.69) *	1.30 (1.01,1.66) *	1.16 (0.87,1.55)
Living alone (Ref. No)	1.63 (1.21,2.19) **	1.33 (1.19,1.99) *	1.64 (1.21,2.23) **	1.55 (1.16,2.07) **	1.28 (0.95,1.72)	1.13 (0.85,1.51)	1.20 (0.84,1.71)
Occupation (Ref. Non-peasant)	1.27 (1.05,1.53) *	1.40 (1.11,1.76) **	1.38 (1.15,1.67) ***	1.44 (1.20,1.73) ***	1.25 (1.03,1.50) *	1.29 (1.07,1.55) **	1.38 (1.11,1.70) **
Enabling variables:							
Financial support sufficient to pay for daily expenses (Ref. No)	1.38 (1.12,1.71) **	1.19 (1.11,1.47) *	1.12 (0.9,1.39)	1.17 (0.95,1.44)	1.15 (0.93,1.43)	1.26 (1.02,1.56) *	1.13 (0.88,1.45)
Total income of your household last year (Ref. <¥15,000)							
¥15,000–50,000	0.78 (0.66,0.93) **	0.71 (0.57,0.88) **	0.89 (0.75,1.06)	0.76 (0.64,0.9) **	0.89 (0.75,1.05)	0.76 (0.64,0.9) **	0.70 (0.57,0.85) ***
>¥50,000	0.86 (0.71,1.05)	0.71 (0.55,0.92) **	0.87 (0.72,1.07)	0.84 (0.69,1.02)	1.14 (0.94,1.40)	0.92 (0.75,1.12)	0.81 (0.64,1.02)
Needs variables:							
Feel lonely and isolated (Ref. Seldom/never)							
Always/often	1.29 (0.93,1.80)	0.93 (0.62,1.39)	1.07 (0.77,1.49)	1.12 (0.81,1.56)	1.04 (0.75,1.45)	1.09 (0.79,1.51)	0.96 (0.66,1.38)
Sometimes	1.48 (1.22,1.79) ***	1.30 (1.01,1.67) *	1.59 (1.3,1.93) ***	1.32 (1.09,1.59) **	1.31 (1.08,1.60) **	1.47 (1.21,1.78) ***	1.51 (1.20,1.91) ***
Feel depressed ^#^ (Ref. No)	1.33 (1.05,1.67)*	1.69 (1.29,2.23) ***	1.36 (1.08,1.72)	1.21 (0.96,1.52)	1.14 (0.9,1.44)	1.31 (1.04,1.65) *	1.42 (1.10,1.84) **
Model summary: Nagelkerke R^2^							
	0.13	0.13	0.12	0.13	0.12	0.12	0.12
χ2 with df = 64 (*p*-value)	115.85(<0.0001)	114.9 (<0.0001)	80.98 (<0.0001)	98.84 (<0.0001)	85.52 (0.0004)	87.95 (0.0002)	89.1 (<0.0001)
−2Log likelihood	4892.38	1414.27	1760.73	1927.03	1766.73	1822.94	1826.54

Note: *n* = 6909. In this study, seven binary logistic regression results were integrated in Table 6, taking “no anticipated needs for community-based services” as a reference. Values in parentheses are the odds ratios without controlling for other variables. The table presents the final results when all sets of variables were entered at once, for the sake of presentational simplification. * *p* < 0.05, ** *p* < 0.01, *** *p* < 0.001.

## Notes

**Ethics Approval and Consent to Participate**: The dataset used in this study is a publicly available dataset. Not applicable.

## Notes

**Availability of Data and Materials**: The CLHLS dataset is publicly available. Information about the data source and available data are found at https://www.icpsr.umich.edu/icpsrweb/DSDR/studies/36179. Researchers can obtain these data after submitting a data use agreement to the CLHLS team.

## Abbreviations

LTC	Long-term Care
CLHLS	Chinese Longitudinal Healthy Longevity Survey
WHO	World Health Organization
